# Supplementary material for: Identification of photoperiod-induced specific miRNAs in the adrenal glands of Sunite sheep (Ovis aries)
Source: Front Vet Sci. 2022 Jul 22;9:888207. doi: 10.3389/fvets.2022.888207 (PMC9354845; doi:10.3389/fvets.2022.888207)
Supplement: Supplementary file 1 [file Data_Sheet_1.ZIP › Supplementary file/miRDeep2 file.zip!/miRDeep2 file/Novel_Structure/LP42A3/Novel_52.pdf]

Provisional ID	:	NC_019469.2_14171
Score total	:	2.2
Score for star read(s)	:	-1.3
Score for read counts	:	0
Score for mfe	:	1.9
Score for randfold	:	1.6
Score for cons. seed	:	
Total read count	:	23
Mature read count	:	21
Loop read count	:	0
Star read count	:	2

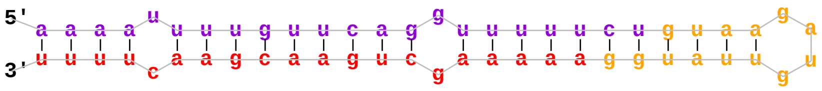

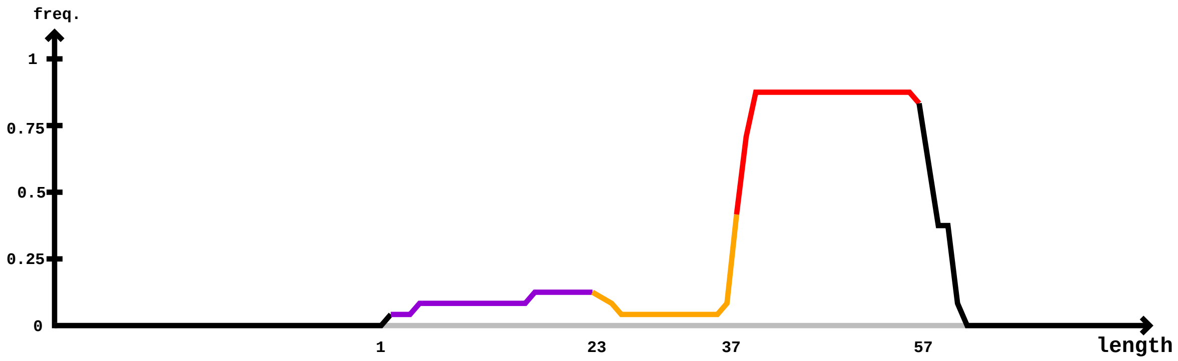

Star Mature

```
5'- ucuuuaggguuuucuuuauguuggguuggcaaaaaaauuuuguucagguuuuucuguaagauguuauggaaaaagcugaacgaacuuuuuggccaacccaguaguaucaugu   -3'    obs
    ucuuuaggguuuucuuuauguuggguuggcaaaaaaauuuuguucagguuuuucuguaagauguuauggaaaaagcugaacgaacuuuuuggccaacccaguaguaucaugu          exp
    ....(((((....)))))..((((((((((..(((((.(((((((((.(((((((((((....))))))))))).))))))))).))))).))))))))))...........    reads     mm      sample
    .................................aaaaGuuuguucagguuuuucu.........................................................    1         1       seq
    ....................................aAuuuguucagguuuuucugu.......................................................    1         1       seq
    ................................................uuuuucuguaGgauguuauggaa.........................................    1         1       seq
    ....................................................................gaaaaaCcugaacgaacuuuu.......................    1         1       seq
    .....................................................................aaaaaCcugaacgaacuuu........................    1         1       seq
    .....................................................................aaaaaCcugaacgaacuuuu.......................    7         1       seq
    ......................................................................aaaaUcugaacgaacuuuu.......................    3         1       seq
    ......................................................................aaaaCcugaacgaacuuuuugg....................    1         1       seq
    ......................................................................aaaagcugaacgaacuuuuugg....................    3         0       seq
    .......................................................................aaagcugaacgaacuuuuugg....................    3         0       seq
    .......................................................................aaagcugaacgaacuuuuuggc...................    2         0       seq
```
